# Do Th17 Lymphocytes and IL-17 Contribute to Parkinson's Disease? A Systematic Review of Available Evidence

**DOI:** 10.3389/fneur.2019.00013

**Published:** 2019-01-24

**Authors:** Elisa Storelli, Niccolò Cassina, Emanuela Rasini, Franca Marino, Marco Cosentino

**Affiliations:** Center of Research in Medical Pharmacology, University of Insubria, Varese, Italy

**Keywords:** Parkinson's disease, Th17 lymphocytes, interleukin-17, neuroinflammation, neurodegeneration, peripheral immunity

## Abstract

Parkinson's disease (PD) is a neurodegenerative disease characterized by progressive loss of dopaminergic neurons, appearance of Lewy bodies and presence of neuroinflammation. No treatments currently exist to prevent PD or delay its progression, and dopaminergic substitution treatments just relieve the consequences of dopaminergic neuron loss. Increasing evidence points to peripheral T lymphocytes as key players in PD, and recently there has been growing interest into the specific role of T helper (Th) 17 lymphocytes. Th17 are a proinflammatory CD4+ T cell lineage named after interleukin (IL)-17, the main cytokine produced by these cells. Th17 are involved in immune-related disease such as psoriasis, rheumatoid arthritis and inflammatory bowel disease, and drugs targeting Th17/IL-17 are currently approved for clinical use in such disease. In the present paper, we first summarized current knowledge about contribution of the peripheral immune system in PD, as well as about the physiopharmacology of Th17 and IL-17 together with its therapeutic relevance. Thereafter, we systematically retrieved and evaluated published evidence about Th17 and IL-17 in PD, to help assessing Th17/IL-17-targeting drugs as potentially novel antiparkinson agents. Critical appraisal of the evidence did not allow to reach definite conclusions: both animal as well as clinical studies are limited, just a few provide mechanistic evidence and none of them investigates the eventual relationship between Th17/IL-17 and clinically relevant endpoints such as disease progression, disability scores, intensity of dopaminergic substitution treatment. Careful assessment of Th17 in PD is anyway a priority, as Th17/IL-17-targeting therapeutics might represent a straightforward opportunity for the unmet needs of PD patients.

## Parkinson's Disease and Peripheral Adaptive Immunity

Parkinson's disease (PD) is a progressive neurodegenerative disease affecting 7 to 10 million people worldwide (1, 2) and is characterized by the progressive loss of dopaminergic neurons in the *substantia nigra pars compacta*, by the appearance of Lewy bodies, which are intracellular inclusions of aggregated α-synuclein, and by the presence of neuroinflammation (3–8). People with PD suffer from motor symptoms, such as bradykinesia, rigidity, resting tremor, and postural instability, as well as from non-motor symptoms, such as autonomic disturbances, depression, and cognitive impairment (9–11). Available therapies are just symptomatic (12), resulting in improved patients' quality of life as disease progresses (13, 14), but unfortunately no treatments exist to prevent or delay PD progression, due to the limited comprehension of the events that lead to neurodegeneration.

Understanding the causes of neurodegeneration in PD remains so far a challenging goal, although novel clues are possibly coming from evidence concerning the role of peripheral adaptive immunity in the regulation of neuroinflammation and neurodegeneration (15–18). Preclinical and epidemiological data strongly suggest that chronic neuroinflammation may slowly bring to neuronal dysfunction during the asymptomatic stage of PD (7, 19). The activation of resident microglia seems to precede dopamine (DA) neuron loss and activators may include interferon (IFN)-γ and tumor necrosis factor (TNF)-α, inducing microglia to commit to a phagocytic activity (1, 20). Activated microglia secretes several neurotoxic substances such as superoxide anions, matrix metalloproteases, nitric oxide, chemokines, proinflammatory cytokines, and glutamate (1, 21). Microglia-derived pro-inflammatory mediators may favor blood-brain barrier (BBB) permeabilisation and subsequent infiltration of peripheral leukocytes into the CNS (22).

Indeed, presence of T lymphocytes has been reported in the *substantia nigra* of parkinsonian brains (20, 23), and both CD8+ and CD4+ T cell subtypes were found in post-mortem brain specimens from PD patients, as well as in animal models of PD (23). CD4+ T lymphocytes are pivotal in the orchestration of an effective immune response during host defense as well as in the pathogenesis of inflammatory diseases. CD4+ T cells may choose either pro-inflammatory phenotypes, such as T helper (Th) 1 and Th17, or anti-inflammatory phenotypes, such as Th2 and the T regulatory (Treg) (24, 25). Interestingly, evidence from both animal models of PD and from clinical studies, suggests that, on one hand, Th1 and Th17 may be detrimental to neurons, and on the other hand, Th2 and Treg may be protective (26, 27).

Understanding whether these cell subsets are imbalanced and how their functions are dysregulated in PD patients could possibly provide novel clues for the understanding of PD pathogenesis and progression as well as for the development of novel therapeutic approaches. Indeed it is now apparently established that in PD patients there is a decreased number of circulating CD4+ T lymphocytes (28), however the relative proportion of CD4+ T cell subsets and their functional profile is still a matter of debate. Our group recently reported that in peripheral blood of PD patients reduction of CD4+ T cells is mostly due to reduced Th2, Th17, Treg, and T naïve cells (29, 30). Consequently Th1 cells, which do not differ between PD patients and healthy subjects in terms of absolute count, are increased with respect to other subsets, leading to a putative Th1 bias, also confirmed by a preferential differentiation of naïve CD4+ T cells of PD patients toward the Th1 lineage and by increased production of IFN-γ and TNF-α (but not of other cytokines, including IL-17) (30). Altogether, such results may not support a role for Th17 in PD, however they are in possible conflict with other studies. For instance, a recent investigation reported increased frequency of Th17 cells in PD patients and a role for IL-17 in T cell-induced cell death of midbrain neurons (31). Since an increasing number of pharmacological agents are being developed targeting IL-17 and Th17 function, we felt mandatory to establish the role—if any—of Th17 cells and IL-17 in neuroinflammation and neurodegeneration occurring in PD, as this would also pave the way for repositioning Th17/IL-17 targeting drugs in PD.

## Overview About Th17 Cells and IL-17

### Physiology and General Pathology of Th17

Th17 have been recognized in 2005 as a distinct lineage and named after IL-17A, which they produce in high amounts (32). Th17 cells function prominently at mucosal surfaces where they trigger pro-inflammatory danger signals that promote clearance of extracellular bacteria and fungi by recruiting and activating neutrophil granulocytes and expressing antimicrobial factors (33, 34). They also directly stimulate the production of mucins (MUC5AC and MUC5) in primary human bronchial epithelial cells *in vitro* (35) as well as the expression of human beta defensin-2 (36) and CC-chemokine ligand 20 (CCL-20) in lung epithelial cells (37).

Th17 cell differentiation is regulated by several transcription factors, including signal transducer and activator of transcription 3 (STAT3), retinoic acid receptor-related orphan receptor-γt (RORγt) and aryl hydrocarbon receptor, and it is driven by transforming growth factor-β (TGF-β), IL-1 and IL-6. IL-23 and TGF-β are critical differentiation factor for Th17 cells. IL-23 (secreted by dendritic cells and tissue-resident macrophages) is required to expand and stabilize the cell population (38). Exposure to this cytokine, after priming with TGF-β and IL-6 (39), is fundamental for functional maturation and pathogenic function of Th17 (40–42). In the absence of inflammation, however, TGF-β alone induces Foxp3 leading to the production of Treg, this way maintaining immune tolerance (43).

Th17 produce several cytokines in addition to IL-17, such as IL-8, IL-21, IL-22, IL-26, TNF-α, granulocyte-monocyte colony stimulatory factor (GM-CSF), CCL20, and IL-10, that allow the recruitment of neutrophils in inflammatory sites (44, 45), even though some of them are not Th17 specific. IL-21 binding to its receptor leads to CD8+ T cell differentiation and proliferation (together with IL-17 and IL-15), B cell differentiation, IL-8 production from dendritic cells and natural killer (NK) cells differentiation (45). IL-22 induces antimicrobial agents and β-defensins in keratinocytes and promotes epidermal hyperplasia (46). Although GM-CSF is not specifically produced by Th17 cells, but also from Th1 cells, it is important in the pathogenicity of Th17 cells in experimental allergic encephalomyelitis (EAE), the animal model of multiple sclerosis (MS), where it seems to induce antigen presenting cells to produce pro-inflammatory cytokines (including IL-6 and IL-23), promoting generation, maturation and survival of Th-17 cells (47–49). Finally, Th17 cells and their secretes are mainly pro-inflammatory and have been linked to several autoimmune diseases such as rheumatoid arthritis (RA), systemic lupus erythematosus (SLE), psoriasis, inflammatory bowel disease (IBD) (45). Evidence however also exists that Th17 may be possibly skewed toward an immune-suppressive regulatory type in a micro-environmental-dependent manner (47, 50). In RA patients there is a high number of IL-17+ and IL-22+ CD4+ T cells in peripheral blood, and IL-17 is present at the sites of inflammatory arthritis, where it amplifies the inflammation induced by other cytokines (45, 51). In patients with SLE there are increased levels of IL-23, IL-21, and IL-17 as a result of the expansion of Th17 cells associated with the depletion of Treg population and increased Th17/Th1 ratio (45, 52). Th1 and Th17 cells infiltrate psoriatic skin lesions, and in particular Th17 determine increased local amounts of IL-17, IL-22, CCL-20, and TNF-α (45, 53). Finally, in IBD patients, high serum concentrations of IL-17 and IL-21 have been reported (45, 54).

### Interleukin-17 Biology and Pharmacology

IL-17A is the founding member of the IL-17 family of cytokines and the major product of Th17 cells. IL-17 family includes six members, designated IL-17A-F. The IL-17 receptor (IL-17R) is expressed ubiquitously, therefore most cells can potentially respond to IL-17 (55). Different IL-17 cytokines have specific receptors (IL-17RA-E). Receptors belonging to the IL-17R family have unique structural features and mediate signaling events that differ from those triggered by other cytokine receptors. All IL-17R subunits are single transmembrane domain-containing proteins with common signaling regions used by at least four ligands (38). The IL-17R complex contains an undetermined number of IL-17RA and IL-17RC subunits, although studies so far indicate that it might be at least trimeric. Both IL-17A and IL-17F signal through these subunits, although IL-17A has far higher affinity for IL-17RA than for IL-17RC, whereas IL-17F has a greater affinity for IL-17RC than for IL-17RA in humans (56). Many cytokine-targeting strategies have been proposed to block signaling through IL-17R, antibodies specific for individual ligands or individual receptor subunits being the most straightforward approach. In addition, soluble IL-17R subunits have been evaluated in pre-clinical models (38).

Among all IL-17 family members, IL-17A and IL-17F are the best characterized. Besides Th17 cells, they are also produced by γδ T cells (57). IL-17 can be produced also by several other innate immune cell types, such as lymphoid tissue inducer cells, natural killer and natural killer T cells, macrophages, Paneth cells (58, 59) and type 3 innate lymphoid cells (ILC-3) (60).

IL-17 immunity has been shown to be essential for muco-cutaneous protection against *Candida albicans* in mice and humans (61), however its dysregulation may cause a variety of disturbances, including autoimmune diseases such as psoriasis and RA, and inflammation-associated cancers such as colorectal carcinoma (62), and blocking IL-17 activity with neutralizing antibodies has emerged as a highly effective therapy for psoriasis and psoriatic arthritis (63). On the other side, in murine models of SLE, deficiency of IL-17 was protective, supporting IL-17 blockade as a potential therapeutic approach in SLE (64).

### Th17 in Autoimmune Disorders of the Nervous System

The contribution of Th17 cells is well established in **MS** (65, 66). In mice with EAE, Th17 cells infiltrate the brains (67) and T cell trafficking to the meninges is supported by ILC-3 cells which produce IL-17, eventually sustaining T cell-induced neuroinflammation and neurodegeneration (60). In MS patients, Th17 frequency, serum levels of IL-17, and IL-17 production by PBMC are higher during relapses (68, 69), and Th17 and Th17-related cytokines may be affected by immunomodulatory therapeutics employed in MS (70–72). Circumstantial evidence also suggests the possible involvement of Th17 in amyotrophic lateral sclerosis, where reports show increased IL-17 and IL-23 serum and cerebrospinal fluid levels (73), as well as increased IL-17 production by cultured peripheral blood mononuclear cells (74). Finally, involvement of Th17 in Alzheimer's disease is suggested by evidence obtained in rodent models (75) as well as in patients (76, 77).

### Pharmacological Modulation of Th17 and IL-17

Additional interest in establishing the possible contribution of Th17 in PD is provided by the increasing opportunities to target the Th17 lineage and its associated cytokines. Several monoclonal antibodies (MoAb) exist which target the IL-17/IL-17R axis (78). While most of them are still in clinical development, the IL-17R-blocking MoAb brodalumab has been recently cleared by FDA for moderate to severe plaque psoriasis (79), and the IL-17-binding MoAb secukinumab has been approved for moderate to severe plaque psoriasis, psoriatic arthritis, and ankylosing spondylitis (80). Despite their efficacy in psoriasis however, brodalumab and secukinumab did not show efficacy (or were even detrimental) in other Th17-related diseases like RA or Crohn's disease [reviewed in Yang et al. (81)], suggesting that Th17 may use mechanisms othr than IL-17 to drive inflammation in different organs and tissues, and/or that targeting the Th17 lineage rather than IL-17 alone could provide better clinical efficacy. Th17-targeting modalities currently under development include small molecule inverse agonists of the Th17 transcription factor RORγt as well as MoAb that block IL-23, which promotes pathogenic Th17 cell function. Ustekinumab is a MoAb targeting the shared IL-12/23 p40 subunit, thus affecting both Th17 and Th1, which received FDA approval for treatment of psoriasis (81). Finally, vitamin D inhibits IL-17 production in rodent and human T cells thus adding to the list of agents potentially affecting Th17 function (82–84).

## Aim

In the present review we systematically retrieved and critically evaluated available evidence regarding the contribution of Th17 cells and IL-17 to neuroinflammation and neurodegeneration in PD, to provide a state-of-the-art compendium which will help identifying the future directions that research in this field may take to assess the possible benefits of targeting Th17/IL-17 to develop novel therapeuticts for PD patients.

## Literature Search Strategy

**Figure 1:**
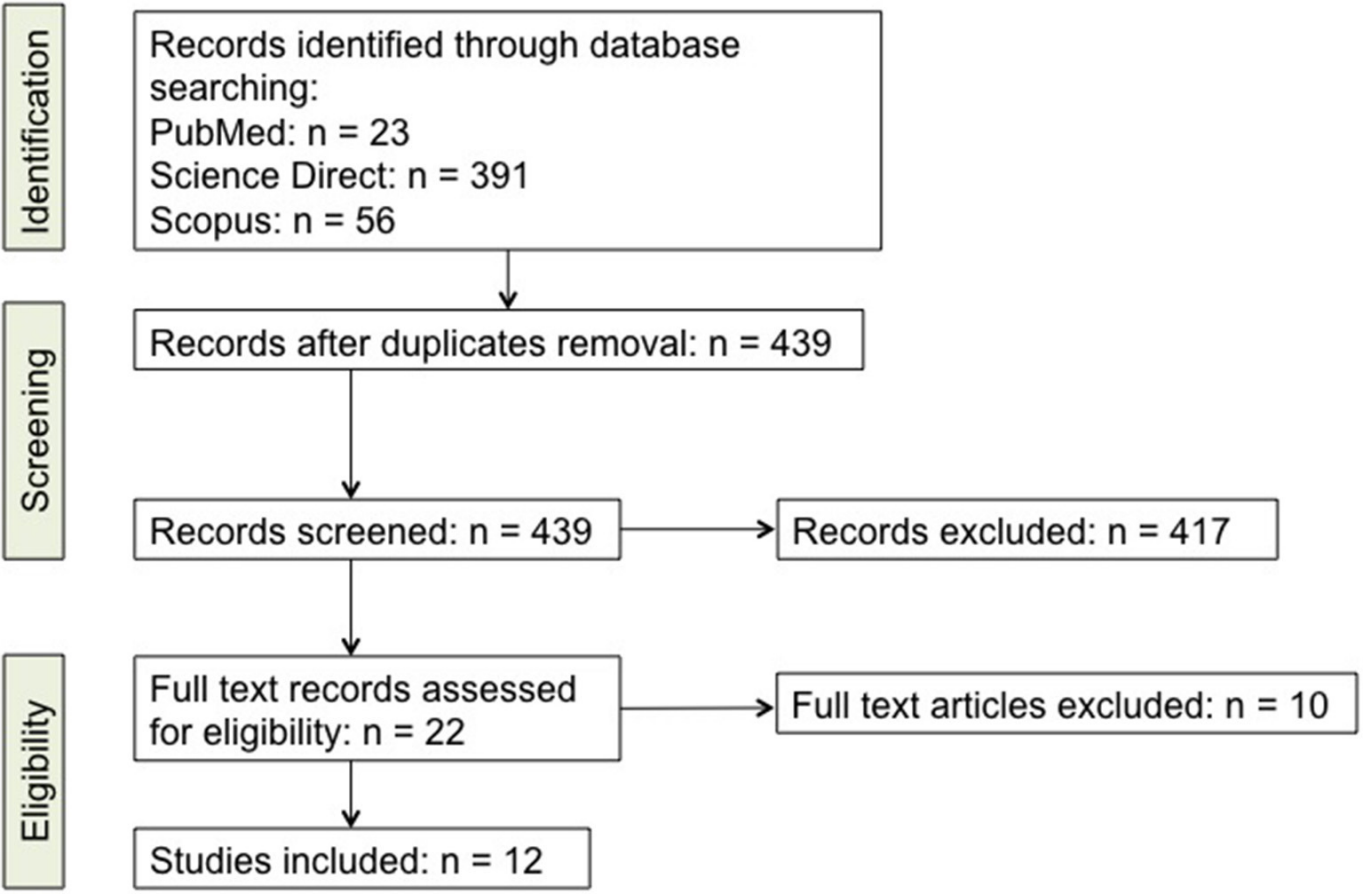
Detailed scheme of the literature screening. A list of the studies is included as Supplementary online data (Supplementary Table 1).

The database analysis for articles selection was done following the PRISMA statement (85). Briefly, literature review through database searching led to a total of 470 reports, including 23 from PubMed, 391 from Science Direct, and 56 from Scopus. For literature retrieval, the following keywords were used: “Th17,” “IL-17,” “Parkinson's Disease,” “PD.” Neither language, nor year restrictions were given, and all reports issued in the period up to and including August 1st, 2018 were included in the screening. After subsequent analysis for relevant titles and abstracts, a total of 417 articles were excluded since they did not specifically focus on PD, Th17 cells or IL-17. Twenty two papers were then selected for full-text eligibility and of those, after full-text reading, 10 were excluded since they were either reviews, or did not deal about PD, Th17 cells and IL-17 together. In the end 12 papers were included in this review. Studies that were considered for inclusion dealt with the changes that Th17 lymphocytes undergo during PD, their effects on neurons in patients and in animal models and the effects/changes of IL-17. A schematic view of the literature selection is given in Figure 1 while the full list of retrieved records is available as Supplementary File (Supplementary Table 1).

## Th17 and IL-17 in PD: a Critical Appraisal of The Evidence

### Animal Studies

We identified 3 studies in animals matching the inclusion criteria, two in C57BL/6J mice treated with i.p. injections of 1-methyl-4-phenyl-1,2,3,6-tetrahydropyridine (MPTP) (86, 87) and one in human *leucine-rich repeat kinase 2* (*LRRK*) *G2019S* gene transgenic rats (88) (Table 1). Both Reynolds et al. (86) and Liu et al. (87) show that MPTP-treated mice have increased Th17 in the *substantia nigra*. Liu et al. (87) reports increased Th17 also in the ventral midbrain. In the first study, immunization of mice with nitrated α-synuclein partially resulted in Th1/Th17 polarization of CD4+ T cells and impaired Treg function, and adoptive transfer of nitrated α-synuclein-primed Th17 cells exacerbated MPTP-induced neurodegeneration while adoptive transfer of Treg was completely protective (86). In the second study, experiments in ventral midbrain cell cultures provided direct evidence and mechanistic explanation of Th17-dependent exacerbation of MPP+-induced dopaminergic cell death (87). As a whole, these two studies provide consistent evidence about the pathogenetic role of Th17 in the MPTP mouse model of PD.

**Table 1:** Evidence from animal models.

**Animals**	**Treatment**	**Main findings**	**References**
C57BL/6J mice	Four i.p. injections of MPTP 16 mg/kg at 2 h intervals.	• MPTP-intoxicated mice had increased infiltration of CD4+ cells (8.5- and 10.3-fold, respectively) within the SN at 7 days-post-intoxication; • immunization of mice with nitrated recombinant α-synuclein (N-4YSyn) partially polarized CD4+ T cells *in vivo* toward either a Th1 or Th17 phenotype, while producing a deficiency in Treg function; • Adoptive transfer of N-4YSyn Th17 cells exacerbated the MPTP-induced loss of striatal TH density; • Adoptive transfer of Treg provided 100% protection of TH+ nigral dopaminergic neurons.	(86)
C57BL/6J mice	Four i.p. injections of MPTP 20 mg/kg at 2 h intervals.	In MPTP-treated mice: • Increased Th17 (identified as CD4/ RORγt-immunoreactive cells) in the SNpc; • Increased Th17 (identified as CD4+IL-17+ cells) in the ventral midbrain; In VM cell cultures, Th17 cells exacerbated MPP+-induced dopaminergic neuronal loss and pro-inflammatory/neurotrophic factor disorders through LFA-1/ICAM-1-mediated Th17-VM neurons communication.	(87)
Human *LRRK2 G2019S* gene transgenic rats	*LRRK2* gene polymorphisms are a risk factor for PD *LRRK2 G2019S* is the most prevalent *LRRK2* mutation found in PD patients.	*LRRK2 G2019S* transgenic rats show: • Decreased numbers of bone marrow myeloid progenitors; • Decreased numbers of Th17 cells in the colon, but not in the brain, during TNBS- or DSS-induced colitis or LPS-induced systemic inflammation; • Myeloid cells defective in supporting Th17 cell differentiation *in vitro*.	(88)

The third selected study does not directly address the impact of Th17 cells of neurodegeneration. Indeed, the animal model consisted of human *LRRK2 G2019S* gene transgenic rats (88) which, as many *LRRK2* transgenic or knockin' rodent models, do not exhibit substantial degeneration of brain dopaminergic neurons [revised in Xiong et al. (89)]. *LRRK2* is one of the key PD-associated genes, which is nonetheless also expressed in immune cells, possibly suggesting a role in immunity and inflammation (90, 91), and in particular the *LRRK2 G2019S* mutation is the most frequent known cause of familial and sporadic PD (92). The study by Park et al. (88) shows that rodents carrying such mutation have decreased numbers of Th17 cells in the colon, unchanged levels of Th17 cells in the brain, ad that myeloid cells are defective in supporting Th17 cell differentiation *in vitro*. While not excluding a neurotoxic potential of Th17 cells, results do not suggest a prominent role by Th17 at least in the *LRRK2 G2019S* mutation-associated form of PD. Indirect support to this conclusion comes from the results of recent studies in mice overexpressing human pathogenic *LRRK2* mutations including *LRRK2 G2019S* (93), showing that brain neuroinflammation was not associated with LRRK2 expression and/or activation in brain microglia nor with myeloid and/or T cell infiltration. Serum cytokines were therefore considered to test the hypothesis that neuroinflammation might be triggered through signaling molecules generated outside the CNS. To this end, wild-type and R1441G mice (but not G2019S mice) were challenged with LPS, showing that no genotype-related differences in cytokine concentration occurred in control conditions, however that after LPS R1441G mice showed increased mRNA levels for several cytokines, most of all IFN-γ, IL-6, IL-10, CCL-5, and M-CSF. Unfortunately, the cytokine panel included none of the main Th17-derived cytokines, however it comprised G-CSF, which can be induced by IL-17 (94). Results showed that after LPS mRNA levels of G-CSF were only slightly different in wild-type and R1441G mice, thus providing no support to the hypothesis of a prominent activation of Th17 cells, in possible agreement with the observation that also CD4+ T cell frequency was not different across the two mouse strains (93).

### Clinical Studies

Literature search retrieved 9 studies which examined Th17 in PD (Table 2). All the studies were performed in sporadic/idiopathic PD patients, compared with healthy subjects (HS) of similar gender distribution and age (although individual matching was actually performed in only one study). In one study (101), subjects were also genotyped for the *LRRK2 G2019S* mutation, however genotypes were used just for patients/HS matching and not for the analysis of Th17 frequency. Five studies, including 193 PD patients and 203 HS, reported increased Th17 frequency in peripheral blood of PD patients, and four, including 215 patients and 165 HS, reported no change or even reduction.

**Table 2:** Evidence from clinical studies.

**Subjects**	**Gender (m/f)**	**Age (years)**	**UPDRS III score**	**H&Y stage**	**Antiparkinson treatment or LED (mg/day)**	**Th17**	**Comment**	**References**
29 patients with sporadic PD vs. 30 HS	PD: 17/12 HS: 16/14	PD: 70.4 ± 7.2HS: 68.9 ± 5.1	23.9 ± 18.5	2.6 ± 0.9	LED: 419.2 ± 237.1	10.0 ± 2.9% in PD patients vs. 11.3 ± 3.2% in HS (*P* > 0.05)	No difference in Th17 frequency between PD patients and HS. PD patients had however lower CD3+, CD4+ T cells, lower Th1 cells, higher B cells and NK cells, while there was no difference in CD+ T cells, Th2, NK-T cells, and Treg.	(95)
45 patients with idiopathic PD vs. 55 HS*^a^*	PD: 28/17 HS: 28/27	Mean (range) PD: 61.4 (40–74) HS: 62(40–70)	Not specified	Range: 1–4	Madopar (levodopa+benserazide)	0.892 ± 0.195% and 0.764 ± 0.151% in PD patients with and without constipation, respectively vs. 0.516 ± 0.157% in HS (*P* < 0.001 in both cases)	Higher Th17 frequency in PD patients in comparison to HS. PD patients had also lower Treg frequency in comparison to HS. Th17 were higher and Treg were lower in PD patients with constipation in comparison to those without constipation.	(96)
40 primary PD patients vs. 40 HS	PD: 22/18 HS: 24/16	PD: 60.4 ± 5.8 HS: 58.6 ± 6.2	Not specified	Not specified	Untreated	0.69 ± 0.11% in PD patients vs. 0.34 ± 0.03% in HS (*P* < 0.001)	Higher Th17 frequency in PD patients in comparison to HS. Compared to HS, PD patients had also lower Treg frequency, lower Treg/Th17 ratio, higher serum IL-17, and similar levels of RORγt mRNA in peripheral blood mononuclear cells.	(97)
64 sporadic PD patients vs. 46 HS	PD: 44/20 HS: 18/28	Not specified	Not specified	Not specified	Not specified	Not shown	No difference in Th17 frequency between PD patients and HS. No difference also in Treg frequency, however when stratified by gender, female PD patients had higher Treg/Th17 ratio in comparison to HS.	(98)
80 initial stage PD patients vs. 80 age/gender matched HS	PD: 40/40 HS: 40/40	PD: 66.2 ± 6.5 HS: 66.7 ± 5.3	13.5 ± 5.3	1.2 ± 0.2	Untreated	1.56 ± 1.38% in PD patients vs. 0.13 ± 0.08% in HS (*P* < 0.001)	Higher Th17 frequency in PD patients in comparison to HS. PD patients had also higher frequency of myeloid-derived suppressor cells (MDSC). Th17 and MDSC showed a positive correlation in PD patients but not in HS.	(99)
18 initial stage PD patients vs. 18 age/gender matched HS	PD: 9/9 HS: 9/9	PD: 68.3 ± 6.0 HS: 64.0 ± 7.0	15.5 ± 6.5	1.1 ± 0.3	Not specified	2.3 ± 1.2 x 10^4^/mL (1.17 ± 0.61%) in PD patients vs. 0.4 ± 0.2 x 10^4^/mL (0.19 ± 0.10%) in HS (*P* < 0.001)	Higher Th17 absolute count and frequency in PD patients in comparison to HS. PD patients had also higher absolute count and frequency of myeloid-derived suppressor cells. Th17 and MDSC showed no correlation either in PD patients or in HS.	(100)
40 PD patients vs. 32 G2019S LRRK2 mutation matched HS	PD: 29/11 HS: 8/24	PD: 67.4 ± 1.3 HS: 65.7 ± 1.9	Not specified	Not specified	Not specified	14.2 ± 1.2% of CD4+ (mean ± SEM) in PD patients vs. 12.9 ± 0.8% in HS *^b^* (*P* > 0.05)	No difference in Th17 frequency between PD patients and HS. PD patients had less CD4+ T cells, however the frequencies of Th1, Th2, Th17 and Treg were the same between PD patients and HS.	(101)
82 idiopathic PD patients (25 drug naive [dn] and 56 on antiparkinson drugs [dt]) vs. 47 HS	PD: 49/33 HS: 25/22	dn: 68.0 ± 9.3 dt: 70.0 ± 8.5 HS:66.8 ± 10.3	dn: 13.6 ± 7.2 dt: 15.3 ± 6.6	dn: 1.3 ± 0.5 dt: 1.9 ± 0.7	11 taking l-DOPA alone, 34 taking l-DOPA + DA agonists without (19) or with rasagiline (15), and 9 taking DA agonists alone (4) or with rasagiline (5). LED: 533.2 ± 360.1	57.1 ± 22.7 x 10^6^/L (8.8 ± 3.9% of CD4+ T cells) in PD-dn patients, 56.9 ± 30.3 x 10^6^/L (7.4 ± 2.5%) in PD-dt patients, and 89.3 ± 48.5 x 10^6^/L (9.2 ± 4.1%) in HS*P* < 0.01 for absolute counts and *P* > 0.05 for % in PD-dn or PD-dt patients vs. HS	Lower Th17 absolute count and no difference in Th17 frequency between PD patients and HS. PD patients had less CD4+ T cells (both as absolute count and as % of total lymphocytes). less Th1/17 and Treg absolute count but same frequency among CD4+ T cells. PD-dt patients had lower Th2 absolute count and frequency and increased Th1 frequency. PD patients had lower levels of RORC mRNA in CD4+ T cells. *Ex vivo* experiments show that: • PD patients CD4+ T cells isolated and cultured for 48 h in resting conditions and activated with PHA produce more IFN-γ and TNF-α (but same amounts of IL-17) in comparison to HS cells; • PD patients naïve CD4+ T cells differentiate more toward Th1 and less toward Th17 in comparison to HS cells.	(30)
10 idiopathic PD patients vs. 10 age/gender matched HS	PD: 9/1 HS: 8/2	PD: 62.5 ± 11.5 HS: 61.9 ± 5.8	14.7 ± 8.3	2.2 ± 0.7	LED: 555.0 ± 325.3	1.67 ± 0.75% of CD3+ T cells in PD patients and 0.75 ± 0.35% in HS (*P* < 0.002)	Higher Th17 absolute count and frequency in PD patients in comparison to HS. *In vitro* experiments show that: • T lymphocytes from PD patients produce more IL-17; • IL-17/IL-17R signaling is involved in T cell-induced cell death of PD patients hiPSC-derived MBNs ^c^.	(31)

*Data are means±SD unless otherwise stated*.

a *Total patients were 102, however only 45 agreed to provide blood samples for T cell subset analysis*.

b *Estimated from Supplementary Figure S3 in Cook et al. (101)*.

c *hiPSC: fibroblast-derived human induced pluripotent stem cells; MBNs: midbrain neurons*.

Several issues must be however considered to compare and interpret results across different studies. First of all, from a methodological point of view the assessment of Th17 cells has been performed by means of substantially different approaches. Three studies (30, 95, 101) assessed Th17 by means of classical phenotypic panels based on the surface markers CXCR3, CCR4 and CCR6 (Table 3) [see e.g., (102)]. On the contrary, the other six studies (31, 96–100) identified Th17 cells by means of IL-17 intracellular staining after short-term cell stimulation with phorbol 12-myristate 13-acetate (PMA)/ionomycin to induce detectable levels of IL-17 [Table 3; see e.g., (103, 104)]. Remarkably, studies identifying Th17 by means of intracellular IL-17 staining reported increased Th17 in PD patients, with the only exception of Cen et al. (98) who found no differences between patients and HS, while studies identifying Th17 cells by means of surface markers found similar or even reduced Th17 in patients in comparison to HS.

**Table 3:** Flow cytometric panels used in clinical studies.

**CD3**	**CD4**	**CD8**	**CD45RO**	**CXCR3**	**CCR4**	**CCR6**	**IL-17A**	**Sample *pretreatment***	**Th17 phenotype**	**Th17 changes in PD patients**	**References**
	x		x			x		Whole blood *n/a*	CD4+/CD45RO+/CCR6+	=	(95)
x	x						x	Whole blood *PMA 2 μg/mL + ionomycin 50 μg/mL*	CD3+/CD4+/IL-17A+	↑	(96)
x		x					x	PBMC *PMA 1 μg/mL + ionomycin 50 μg/mL*	CD3+/CD8-/IL-17A+	↑	(97)
x		x					x	PBMC *leukocyte activator cocktail (?)*	CD3+/CD8-/IL-17A+	=	(98)
x		x					x	PBMC *PMA 50 ng/mL + ionomycin 500 ng/mL*	CD3+/CD8-/IL-17A+	↑	(99)
x		x					x	PBMC *PMA 50 ng/mL + ionomycin 500 ng/mL*	CD3+/CD8-/IL-17A+	↑	(100)
x	x		x	x		x		PBMC *n/a*	CD3+/CD4+/CD45RO+/ CXCR3-/CCR6+	=	(101)
	x			x	x	x		whole blood *n/a*	CD4+/CXCR3-/CCR4+/CCR6+	= /↓	(30)
	x						x	CD3+ T cells *PMA 20 ng/mL + ionomycin 1 μM*	CD4+/IL-17A+	↑	(31)

*n/a, not applicable; PMA, phorbol 12-myristate 13-acetate; PBMC, peripheral blood mononuclear cells; = : unchanged; ↑: increased; ↓: reduced*.

Another critical issue is that in most of the studies Th17 are quantified just as percentage of a reference cell population and not as absolute number of cells per volume of blood. The only two studies providing this latter information are Chen et al. (100) and Kustrimovic et al. (30). The former, in which Th17 were found apparently increased also as absolute numbers, however did not actually perform a direct count of Th17 but just obtained an absolute number using percentages. In other terms, they did not report the actual number of Th17 cells per volume of blood but rather subsequently referred to blood the percentage of IL-17+ cells found in cultured PBMC after separation from whole blood and stimulation with PMA/ionomycin. Kustrimovic et al. (30) on the contrary reported absolute numbers referred to whole blood, and found reduced Th17 in PD patients. This finding is in keeping with the decreased numbers of CD3+ and CD4+ T lymphocytes which have been consistently reported in peripheral blood of PD patients across several studies (28), and which according to Kustrimovic et al. (30) are likely due to decreased Th2, Th17, and Treg. It cannot be excluded therefore that percentage increase of IL17+ T cells may occur in a context where the absolute amounts of circulating Th17 cells is actually decreased. Would this be the case, then direct assessment of IL-17 released from T cells and/or present in serum or tissues becomes of critical importance to establish whether increased Th17/IL-17 function is really enhanced.

Unfortunately, data on IL-17 production by T cells have been provided so far only by Sommer et al. (31) and Kustrimovic et al. (30). In the first study, IL-17 was measured in supernatants after 3-days co-culture of human induced pluripotent stem cells (hiPSCs)-derived neurons with autologous T lymphocytes from 3 patients and 3 HS, finding increased amounts of IL-17 in the supernatants of PD co-cultures. The study also includes pharmacological experiments suggesting the involvement of IL-17/IL-17R signaling in T cell-induced cell death of PD patients' hiPSC-derived mesencephalic brain neurons (31). In the second study, IL-17 was assayed in the supernatants of CD4+ T cells cultured for 48 h in resting conditions and activated with phytohaemagglutinin (PHA). Cells from 6 PD patients on antiparkinson drugs, 4 PD patients who never received dopaminergic substitution treatment and 4 HS were tested, and no difference was observed in IL-17 production either in resting conditions or after PHA, while other cytokines such as IFN-γ and TNF-α were hugely increased in cells from patients (30). The possible contribution of differences in the experimental models used in these studies in the discrepancy regarding IL-17 production by T cells of PD patients remains an unresolved issue.

In summary, while the frequency of Th17 identified by means of their established surface markers may not differ between PD patients and controls, increased frequency of IL-17-producing cells has been consistently reported. *Ex vivo* evidence showing increased secretion of IL-17 from T cells of PD patients is however scarce and contradictory, as only two studies exist which used profoundly different experimental models, one showing enhanced production in samples from just 3 patients in comparison to 3 controls (31) and another one showing no difference in 11 patients in comparison to 4 controls (30). Last but not least, no studies found correlations between Th17/IL-17 and clinically relevant measures of disease state and/or progression, such as disease duration, disability scores, intensity of dopaminergic substitution therapy, etc. Clinical evidence regarding dysregulated frequency and/or function of Th17 cells in PD patients remains therefore scarce and inconclusive.

## Discussion

Evidence about the involvement of Th17 in PD is so far limited and controversial, as preclinical studies in animal models are just a few while clinical studies provide inconclusive results. While the involvement of Th17 in the MPTP mouse model of PD has been confirmed by two studies (86, 87), no data exist in other models, based either on treatment of animals with different neurotoxins or on genetic modifications. Indeed, neurotoxin-based models are best recapitulating nigrostriatal degeneration, but for instance MPTP-treated mice do not exhibit Lewy bodies and/or α-synuclein-like pathology, while genetic models may better simulate genetic forms of PD (105). The only other animal study available was based on human *LRRK2 G2019S* gene transgenic rats, which however show only mild behavioral alterations and no brain damage (88). Remarkably, in this study the presence of *LRRK2 G2019S*, one of the major PD-linked gene mutations, was associated with unchanged brain Th17 cells and even decreased Th17 in periphery, as well as with defective myeloid cells-induced Th17 differentiation *in vitro*.

On the other side, only few clinical studies included some mechanistic evidence in addition to the mere enumeration of circulating Th17. Possibly, the main apparently conflictual evidence arising is that Th17 are not different between PD patients and controls when assessed by means of Th17-associated surface markers, while they are increased if assayed as IL-17+ cells (Tables 2, 3). Th17 subsets identified by these two methods indeed do not coincide. Evidence exists that Th17-associated surface markers—and in particular CCR6—are expressed on virtually all IL-17+ T lymphocytes. IL-17 is however usually undetectable in freshly isolated cells, which require activation to express measurable IL-17 (as in 5 out of the 6 studies which used intracellular IL-17 staining to identify Th17 cells; Table 3). Following activation however IL-17+ T cells may usually represent on average just 7–8% of total Th17 cells, depending on the specific protocol of isolation, culture, and stimulation of T cells (106, 107), although at least a few studies exist showing that they may correlate with Th17 frequency assessed by means of surface markers (108).

A provisional interpretation which might possibly reconcile available evidence is that PD patients have similar percentages of Th17 cells, which however may harbor an increased proportion of cells ready to express IL-17 upon activation. Whether this increased percentage of IL-17-producing cells may actually result in increased Th17/IL-17 activity remains however to be established, in view of the limited and conflicting evidence about IL-17 amounts actually secreted by PD patients lymphocytes, which is unchanged according to Kustrimovic et al. (30) and increased based on Sommer et al. (31), both unfortunately performing experiments in cell preparations from very few subjects. In addition, Kustrimovic et al. (30) reported reduced absolute numbers of Th17 cells per volume of blood in PD patients, thus highlighting the possibility that even increased percentages of IL-17-producing cells might not necessarily result in enhanced Th17/IL-17-dependent systemic proinflammatory effects.

As a whole, evidence about Th17 contribution in PD remains thus circumstantial and awaits further confirmation and in-depth investigation. Before driving provisional conclusions from available studies directly addressing Th17 and IL-17 in PD patients and in animal models of the disease, a few additional indirect lines of evidence about Th17/IL-17 and PD deserve consideration to put the issue in full context, namely: (i) the correlation between PD and immune-related disease, (ii) recent studies about gut microbiome in PD and (iii) on vitamin D, and (iv) emerging evidence regarding dopaminergic modulation of Th17 cells and (v) the influence of Th17 on glial cells.

### PD and Immune-Related Disease

There is extensive literature about the association between PD and immune-related disease such as autoimmune disease and cancer. Epidemiological studies strongly suggest that PD patients are at lower risk for most cancers, with the notable exception of breast cancer and melanomas, which may occur more frequently in PD patients as compared with controls (109). Th1 lymphocytes are responsible for cell-mediated immunity to intracellular pathogens and tumor cells (110), thus a relative increase in Th1 in PD patients [as reported by Kustrimovic et al. (30)] might well contribute to lower susceptibility to cancers. Such hypothesis is further supported by evidence in PD patients regarding increased CD4+ T cell production of IFN-γ and TNF-α, two Th1 cytokines critical for antitumor immunity, and their insensitivity to Treg, as well as by the preferential differentiation of naive CD4+ T cells from PD patients toward the Th1 lineage (30). Th17 cells on the contrary play controversial roles in antitumor immunity (111), nonetheless, at least in mice Th17 cells eradicate melanoma tumors to a greater extent than Th1 cells (112). Therefore, increased frequency of melanomas in PD might eventually stand against a systemic increase of Th17 activity in PD.

Immune-related disease associated with PD also include psoriasis, IBD and RA. Patients with psoriasis may have on average 38% increased risk to develop PD, possibly as a result of chronic inflammation (113). The relationship between PD and IBD is much more controversial, since although gene association studies suggest that functional variants in the *LRRK2* gene may confer shared effects on risk for Crohn's disease and PD, it has been also reported that PD as a whole is associated with lower risk of IBD (−15%) as well as of Crohn's disease (−17%) and ulcerative colitis (−12%) (114). Another study however showed that IBD may increase by 28% the risk of developing PD, while treatment of IBD with anti-TNF-α therapy reduced PD incidence rate among IBD patients by 78% (115). **RA** as well may be associated with a reduced risk of developing PD (−35%) (116). It must be also considered however that in other population-based studies no association between PD and autoimmune disease has been reported, with the only remarkable exception in rheumathoid arthritis patients of a reduced risk of developing PD (−30%, (117)). Psoriasis, IBD and RA are however complex diseases currently seen as supported by both Th1 and Th17-mediated inflammation (118–120), thus evidence for association of any of these immune-mediated diseases with PD provides just a few if any contribution to unravel the specific role of Th17 in PD.

### Clues From Gut Microbiome Studies

The gut microbiome attracts increasing attention as key regulator of brain development and homeostasis, possibly acting also through the immune system (121), and as a consequence the gut microbiome in PD represents a rapidly growing area of research. A recent study compared the fecal microbiomes of PD patients and controls, showing in feces of PD patients nearly 80% decreased abundance of *Prevotellaceae*, and increased abundance of other families including *Lactobacillaceae* (122). *Prevotellaceae* abundance may be associated with increased Th17-mediated mucosal inflammation (123), while *Lactobacillaceae* may induce Th1-type immune responses (124). Modifications of the intestinal microbiome in PD might be thus related to reduced Th17 cells and the Th1-biased immunity which we found in PD patients (30).

### Th17, Vitamin D, and PD

Vitamin D exerts direct and indirect effects on T lymphocytes, and its deficiency has been linked to autoimmune and cardiovascular disease, hypertension, and even cancer. Remarkably, in both rodent and human T cells vitamin D has been shown to inhibit production of IL-17 and IFN-γ and to promote Treg differentiation and function (82–84). Several lines of evidence suggest that PD patients may be low in vitamin D and that vitamin D supplementation prevents dopaminergic neuron loss in animal models and may be beneficial in patients (125, 126). It cannot be excluded that at least part of vitamin D-induced benefits in PD might depend on its immune effects. Studies are needed however to assess the eventual occurrence and the relative contribution of vitamin D-dependent modulation of Th1, Th17, and Treg in PD.

### Dopaminergic Modulation of Human Th17

No therapies are available for PD, and symptomatic treatments rely on dopamine substitution treatments (including the DA precursor l-DOPA, DA agonists and indirect dopaminergic agents) (12). Besides its role as neurotransmitter, DA is however a key transmitter between the nervous and the immune system as well as among immune cells and peripheral tissues (127–129). Although information on DA and Th17/IL-17 is still limited and fragmentary, in rodent models it has been shown that D_1_-like D5 DR expressed on DC may contribute to Th17 differentiation and severity of EAE (130). Recently, Melnikov et al. (69) reported that DA may inhibit IL-17 and IFN-γ production in cultured peripheral blood mononuclear cells from HS and MS patients. The ability of DA to inhibit IFN-γ production in peripheral blood mononuclear cells (PBMC) was previously reported by Ferreira et al. (131), who however did not observe any effect of DA on IL-17 or on the Th17-related cytokines. Remarkably, in a subsequent study Ferreira et al. (132) confirmed no effect by DA on IL-17 produced by PBMC but, in apparent contrast with Melnikov et al. (69), reported the ability of DA to increase IL-17 from cells of MS patients. No evidence was provided about the receptor pathways involved, anyway such observations highlight the need for further studies. Melnikov et al. (69) showed that in their experiments the inhibitory effect of DA on IL-17 was antagonized by the D_2_-like DR antagonist sulpiride but not by the D_1_-like DR antagonist SCH-23390. Unfortunately, pharmacological experiments were not performed on IFN-γ production. Such results deserve careful consideration for their potential implications concerning PD. Indeed, DA agonists used as antiparkinson drugs act mainly on D_2_-like DR (for instance, rotigotine is a D_1_-like/D_2_-like DR mixed agonist, while pramipexole is a D_2_-like DR agonist). Would ropinirole or pramipexole modulate IL-17 and IFN-γ production by T cells of PD patients at therapeutically relevant concentrations, DA agonists in PD could possibly shift from mere DA substitutes to immunomodulating drugs targeting Th17 and Th1 mechanisms potentially involved in neuroinflammation underlying PD neurodegeneration.

### Influence of Th17 on Glial Cells

The pathogenic role of Th17 cells in the CNS has received extensive attention on the pathogenesis of autoimmune demyelinating diseases, where their contribution is well established (133), and at least one proof-of-concept study provided evidence that secukinumab, which blocks IL-17A, may reduce lesions in MS patients (134). In this context, the ability of Th17 cells to affect glia has received specific attention. Evidence in animal models of MS suggests that Th17 cells preferentially affect astrocytes rather than microglia. In integrin α4-deficient mice, where trafficking of Th1 but not Th17 cells into the CNS is compromised, induction of experimental autoimmune encephalomyelitis results in microglial activation but comparable astrogliosis in comparison to wild-type mice (135). Indeed, while Th1-derived secretions trigger proinflammatory responses in microglia, neither Th17-derived secretions nor increased expression of IL-17A in the brain apparently affect microglial function (135). While miroglia has an established role in neuroinflammation and neurodegeneration in PD (136), the involvement of astrocytes is still a matter of debate (137). As for IL-17, it has been shown that exposure of microglia to IL-17A results in activation and increased production of proinflammatory cytokines, and IL-17A-neutralizing antibodies prevented neuroinflammation and cognitive impairment in rodents (138). On the other side, at least *in vitro* TLR-dependent activation of microglia has been shown to polarize γδ T cells toward neurotoxic IL-17+ γδ T cells (139).

## Conclusions

Critical appraisal of evidence retrieved after a systematic revision of the literature available about Th17/IL-17 and PD do not allow to reach definite conclusions. Both animal, as well as clinical studies, are limited and in particular the latter are mainly concerned with Th17 frequency rather than function and relationship with PD pathology and clinical progression.

On the other side, indirect evidence potentially may stand in favor of a contribution of Th17 in PD (as suggested by studies on vitamin D) but also against it (in the case of recent studies addressing gut microbiome in PD patients), or may be also once more conflicting (such as studies correlating PD with immune-related disease).

Future research on Th17 in PD patients should thus first of all thoroughly assess circulating Th17 using both surface markers and intracellular IL-17 staining, considering not only cell frequency but also absolute numbers per volume of blood. It would be also necessary to include investigation of IL-17 (as well as of other Th17-related cytokines) secretion and serum/tissue levels.

**Figure 2:**
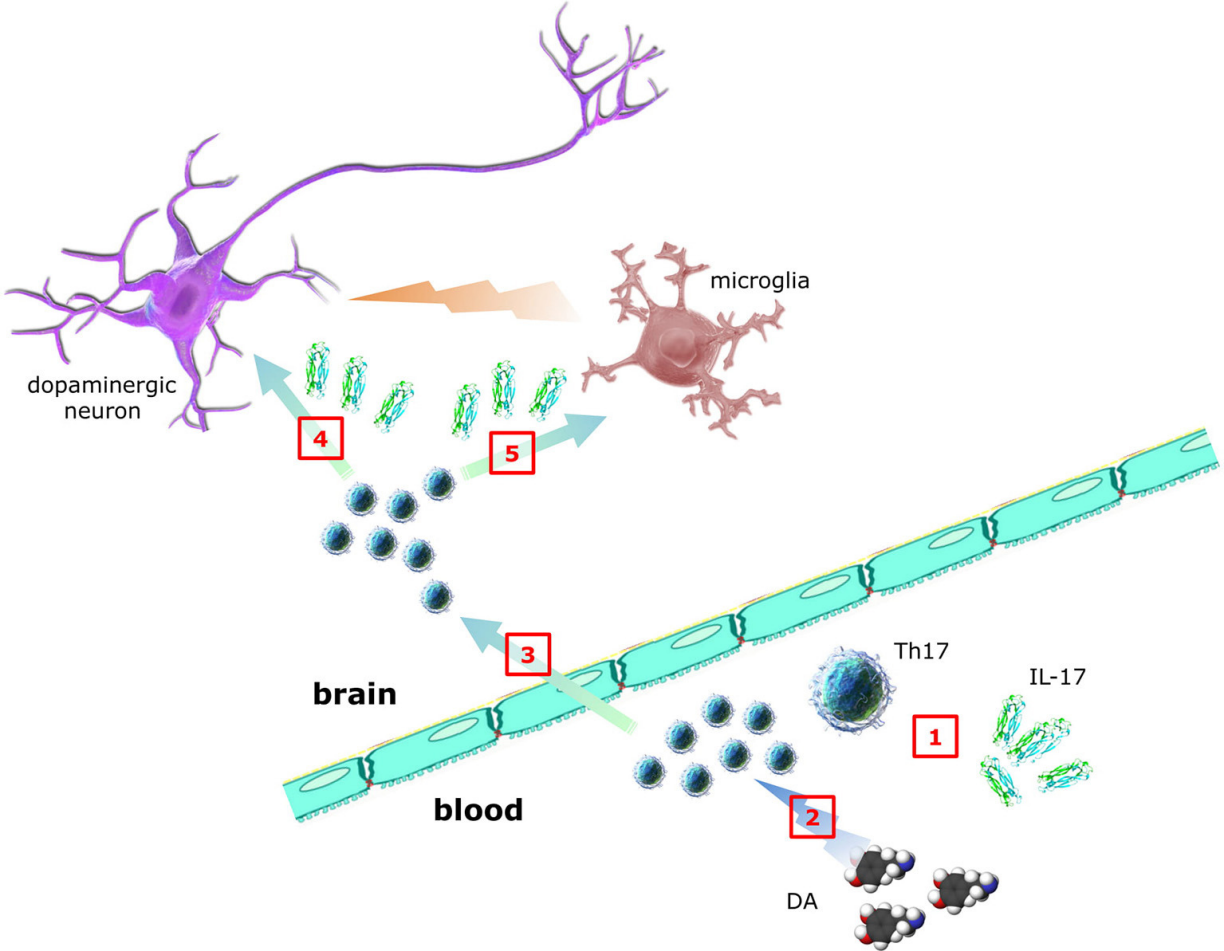
Contribution of Th17 lymphocytes and IL-17 to PD. Whether Th17 and IL-17 in peripheral blood of PD patients (1) are increased, decreased or unchanged is still debated, despite many studies addressed the issue. DA itself may also affect Th17 function, however whether dopaminergic substitution therapy results in any Th17/IL-17 changes is presently unknown (2). In addition, although it is established that T cells infiltrate brains of PD patients, direct demonstration of Th17 has not yet been provided (3). In the same way, although *in vitro* Th17/IL-17 have been shown to exert neurotoxic effects, the clinical relevance of such observations awaits confirmation (4). Finally, despite circumstantial evidence suggesting Th17-glial cells interplay, no data exist so far in PD (5) (individual parts of the figure have been taken and modified from the Wikimedia Commons - http://commons.wikimedia.org).

As suggested in Figure 2, key questions to be answered include:

1. Which is the relationship between Th17/IL-17 and PD pathology and clinics (such as disease duration, disability scores, intensity of dopaminergic substitution therapy, etc.)?
2. Which are the mechanisms and the cellular targets (including neurons, microglia, and astrocytes) underlying Th17/IL-17 contribution to PD pathogenesis and progression (in this regard the study by Sommer et al. (31) may be a primer, however results await reproduction and extension)?
3. Do dopaminergic agonists currently used in PD therapeutics affect Th17/IL-17, as reported by *in vitro* evidence for D2-like DR-dependent modulation of human Th17 (69)?

In this last regard, it should be taken in mind that therapy with dopaminergic agonists (and possibly with l-DOPA and other indirect dopaminergic agents) could be also a confounding factor in studies of Th17/IL-17 in PD patients.

Careful assessment of Th17 in PD is anyway a priority in the context of the emerging area of peripheral immunity in PD, also in view of the increasing number of therapeutics targeting Th17/IL-17 pathways approved for clinical indications and which might therefore represent potentially novel antiparkinson drugs.

## Author Contributions

FM and MC: study conception and design. ES, NC, and ER: acquisition of data. ES, NC, ER, FM, and MC: analysis and interpretation of data. All authors were involved in drafting the article or revising it critically for important intellectual content, and all authors approved the final version to be published. All authors agree to be accountable for all aspects of the work in ensuring that questions related to the accuracy or integrity of any part of the work are appropriately investigated and resolved, and declare to have confidence in the integrity of the contributions of their co-authors.

### Conflict of Interest Statement

The authors declare that the research was conducted in the absence of any commercial or financial relationships that could be construed as a potential conflict of interest.
